# How Does ChatGPT Perform on the United States Medical Licensing Examination (USMLE)? The Implications of Large Language Models for Medical Education and Knowledge Assessment

**DOI:** 10.2196/45312

**Published:** 2023-02-08

**Authors:** Aidan Gilson, Conrad W Safranek, Thomas Huang, Vimig Socrates, Ling Chi, Richard Andrew Taylor, David Chartash

**Affiliations:** 1 Section for Biomedical Informatics and Data Science Yale University School of Medicine New Haven, CT United States; 2 Department of Emergency Medicine Yale University School of Medicine New Haven, CT United States; 3 Program of Computational Biology and Bioinformatics Yale University New Haven, CT United States; 4 School of Medicine University College Dublin National University of Ireland, Dublin Dublin Ireland

**Keywords:** natural language processing, NLP, MedQA, generative pre-trained transformer, GPT, medical education, chatbot, artificial intelligence, education technology, ChatGPT, conversational agent, machine learning, USMLE

## Abstract

**Background:**

Chat Generative Pre-trained Transformer (ChatGPT) is a 175-billion-parameter natural language processing model that can generate conversation-style responses to user input.

**Objective:**

This study aimed to evaluate the performance of ChatGPT on questions within the scope of the United States Medical Licensing Examination (USMLE) Step 1 and Step 2 exams, as well as to analyze responses for user interpretability.

**Methods:**

We used 2 sets of multiple-choice questions to evaluate ChatGPT’s performance, each with questions pertaining to Step 1 and Step 2. The first set was derived from AMBOSS, a commonly used question bank for medical students, which also provides statistics on question difficulty and the performance on an exam relative to the user base. The second set was the National Board of Medical Examiners (NBME) free 120 questions. ChatGPT’s performance was compared to 2 other large language models, GPT-3 and InstructGPT. The text output of each ChatGPT response was evaluated across 3 qualitative metrics: logical justification of the answer selected, presence of information internal to the question, and presence of information external to the question.

**Results:**

Of the 4 data sets, *AMBOSS-Step1*, *AMBOSS-Step2*, *NBME-Free-Step1*, and *NBME-Free-Step2*, ChatGPT achieved accuracies of 44% (44/100), 42% (42/100), 64.4% (56/87), and 57.8% (59/102), respectively. ChatGPT outperformed InstructGPT by 8.15% on average across all data sets, and GPT-3 performed similarly to random chance. The model demonstrated a significant decrease in performance as question difficulty increased (*P*=.01) within the *AMBOSS-Step1* data set. We found that logical justification for ChatGPT’s answer selection was present in 100% of outputs of the *NBME* data sets. Internal information to the question was present in 96.8% (183/189) of all questions. The presence of information external to the question was 44.5% and 27% lower for incorrect answers relative to correct answers on the *NBME-Free-Step1* (*P*<.001) and *NBME-Free-Step2* (*P*=.001) data sets, respectively.

**Conclusions:**

ChatGPT marks a significant improvement in natural language processing models on the tasks of medical question answering. By performing at a greater than 60% threshold on the *NBME-Free-Step-1* data set, we show that the model achieves the equivalent of a passing score for a third-year medical student. Additionally, we highlight ChatGPT’s capacity to provide logic and informational context across the majority of answers. These facts taken together make a compelling case for the potential applications of ChatGPT as an interactive medical education tool to support learning.

## Introduction

Chat Generative Pre-trained Transformer (ChatGPT) [1] is a 175-billion-parameter natural language processing model that uses deep learning algorithms trained on vast amounts of data to generate human-like responses to user prompts [2]. As a general purpose dialogic agent, ChatGPT is designed to be able to respond to a wide range of topics, potentially making it a useful tool for customer service, chatbots, and a host of other applications. Since its release, it has garnered significant press for both seemingly incredible feats such as automated generation of responses in the style of Shakespearean sonnets while also failing to answer simple mathematical questions [3-5].

ChatGPT is the latest among a class of large language models (LLMs) known as autoregressive language models [6]. Generative LLMs believed to be similar to ChatGPT are trained using the decoder component of a transformer model [7], tasked with predicting the next token in a sequence on large corpora of text [8-10]. Such foundation models are often fine-tuned on task-specific data to improve performance. However, the introduction of OpenAI’s GPT-3 presented the first in a line of highly scaled LLMs that achieve state-of-the-art performance with little fine-tuning required [6]. ChatGPT builds on OpenAI’s previous GPT-3.5 language models with the addition of both supervised and reinforcement learning techniques [1]. ChatGPT is a direct descendant of InstructGPT, a fine-tuned version of GPT-3.5 trained on human-derived responses to prompts submitted to the OpenAI application programming interface (API) Playground. InstructGPT was developed by first being tasked to generate a set of responses to a particular prompt and having human annotators label the preferred answer. These preferences are then maximized in a reward model trained using Proximal Policy Optimization, a reinforcement learning algorithm, to tune InstructGPT. ChatGPT is reported to be specifically trained on conversational prompts to encourage dialogic output.

Within the medical domain, LLMs have been investigated as tools for personalized patient interaction and consumer health education [11,12]. Although demonstrating potential, these models have had limited success testing clinical knowledge through (generative) question-answering tasks [13,14]. ChatGPT could represent the first in a new line of models that may better represent the combination of clinical knowledge and dialogic interaction. ChatGPT’s interface that produces unique narrative replies allows for novel use cases, such as acting as a simulated patient, a brainstorming tool providing individual feedback, or a fellow classmate to simulate small group–style learning. For these applications to be useful, however, ChatGPT must perform comparably to humans on assessments of medical knowledge and reasoning such that users have sufficient confidence in its responses.

In this paper, we aimed to quantify ChatGPT’s performance on examinations that seek to assess the primary competency of medical knowledge—established and evolving biomedical, clinical, epidemiological, and social-behavioral science knowledge—and a facet of its application to patient care through the use of 2 data sets centered around knowledge tested in the United States Medical Licensing Examination (USMLE) Step 1 and Step 2 Clinical Knowledge exams. Step 1 focuses on foundational sciences and their relation to the practice of medicine, whereas Step 2 focuses on the clinical application of those foundational sciences. USMLE Step 3 was excluded as it is intended to assess skills and capacity for independent generalist medical practice rather than foundational knowledge. We also compared the performance of ChatGPT on these examinations to the performances of 2 previously mentioned LLMs, GPT-3 and InstructGPT. In addition, to further assess the ability of ChatGPT to serve as a simulated medical tutor, we qualitatively examined the integrity of ChatGPT’s responses with regard to logical justification and the use of intrinsic and extrinsic information.

## Methods

### Medical Education Data Sets

We created 2 pairs of data sets to examine ChatGPT’s understanding of medical knowledge related to Step 1 and Step 2. We first selected a subset of 100 questions from AMBOSS, a widely used question bank that contains over 2700 Step 1 and 3150 Step 2 questions [15]. The existing performance statistics from previous AMBOSS users allows us to determine the relative performance of the model. We call these data sets *AMBOSS-Step1* and *AMBOSS-Step2*. AMBOSS provides users with an *Attending Tip* when they have difficulty with a question, as well as a difficulty rating (1-5). We included a second instance of each question including these tips in our data set to determine if the additional context provided by the tip improves performance.

We also used the list of 120 free Step 1 and Step 2 Clinical Knowledge questions developed by the National Board of Medical Examiners (NBME), which we call *NBME-Free-Step1* and *NBME-Free-Step2*, respectively, to evaluate ChatGPT’s performance on the questions most closely aligned with those from the true licensure exams.

### Prompt Engineering

**Figure 1:**
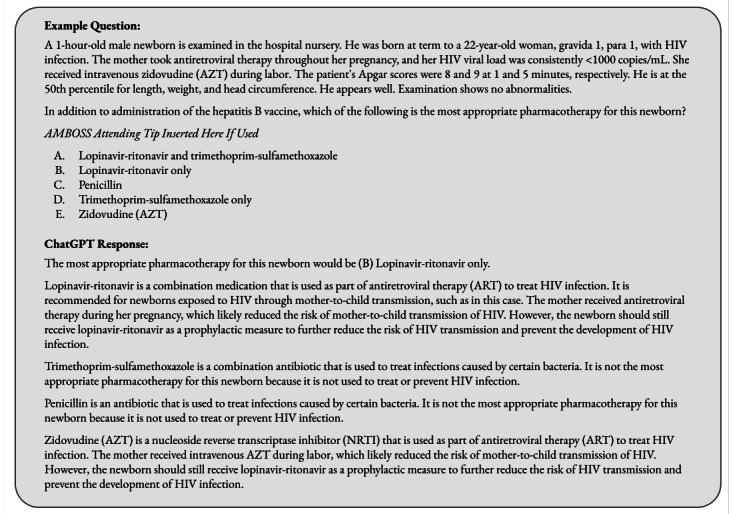
Template of question posed to each large language model (LLM), including both AMBOSS *Attending Tip* and the response from Chat Generative Pre-trained Transformer (ChatGPT). The correct answer to this question is “E. Zidovudine (AZT).” In the case of GPT-3, prompt engineering was necessary, with: "Please answer this multiple choice question:" + question as described previously + "Correct answer is." As GPT-3 is inherently a nondialogic model, this was necessary to reduce model hallucinations and force a clear answer [17].

Due to the significant impact that prompt engineering has been shown to have on generative LLM output, we standardized the input formats of the *AMBOSS* and *NBME* data sets [16]. First, we removed any questions that include an image, as ChatGPT only accepts textual input. Next, we removed questions where the answer was formatted as a table. This was done so that the accuracy of ChatGPT’s answers was solely dependent on its ability to synthesize medical knowledge within narrative text rather than parsing complicated text inputs. Questions were formatted with the question text followed by the direct question separated by a new line. In the *AMBOSS* data sets, the *Attending Tip* was inserted as a separate instance of the question. Following the question text and direct question, the multiple-choice answers were provided, separated again by a new line. An example question prompt and response is shown in Figure 1.

### Model Testing

We first recorded all correct answers as they appeared in the *AMBOSS* and *NBME* data sets. All model testing was performed on the December 15, 2022, version of ChatGPT by manually entering questions into the ChatGPT website. The OpenAI API was used to query GPT-3 and InstructGPT using the *davinci* and *text-davinci-003* models, respectively. We then prompted the models with the standardized questions. We also further prompted ChatGPT with questions including the *Attending Tip*. All responses were directly copied into a shared spreadsheet for review. Due to the nature of each model’s output, we manually reviewed each answer to determine which answer from the multiple-choice question was selected, if any.

We then qualified the ChatGPT responses for each question using 3 binary variables characteristic of narrative coherence [18]. Without deeper linguistic analysis, these variables provide a crude metric, assessing the following:

1. Logical reasoning: The response clearly identifies the logic in selecting between answers given the information presented in the response.
2. Internal information: The response uses information internal to the question, including information about the question in the response.
3. External information: The response uses information external to the question, including but not limited to qualifying the answers given or the stem.

Finally, for each question answered incorrectly, we labeled the reason for the incorrect answer as one of the following options:

- Logical error: The response adequately found the pertinent information but did not properly convert the information to an answer.
  Example: Identifies that a young woman has been having difficulty with taking pills routinely and still recommends oral contraceptives over an intrauterine device.
- Information error: ChatGPT either did not identify a key piece of information, whether present in the question stem or through external information, that would be considered expected knowledge.
  Example: Recommends antibiotics for sinusitis infection, believing most cases to be of bacterial etiology even when the majority are viral.
- Statistical error: An error centered around an arithmetic mistake. This includes explicit errors, such as stating “1 + 1 = 3,” or indirect errors, such as an incorrect estimation of disease prevalence.
  Example: Identifies underlying nephrolithiasis but misclassifies the prevalence of different stone types.

All authors who performed qualitative analysis of the responses (AG, CWS, RAT, and DC) worked collaboratively, and all uncertain labels were reconciled.

### Data Analysis

All analysis was conducted in Python software (version 3.10.2; Python Software Foundation). Unpaired chi-square tests were used to determine whether question difficulty significantly affected ChatGPT’s performance on the *AMBOSS-Step1* and *AMBOSS-Step2* data sets*.* Similarly, unpaired chi-square tests were also used to evaluate the distribution of logical reasoning, internal information, and external information between correct and incorrect responses in the *NBME-Free-Step1* and *NBME-Free-Step2* data sets.

## Results

### Overall Performance

Table 1 shows the performance of 3 LLMs: ChatGPT, GPT-3, and InstructGPT, on the 4 data sets tested. Scores for *AMBOSS* models are shown when the *Attending Tip* was not used. ChatGPT performed more accurately on Step 1 related questions compared to Step 2 questions on both the *NBME* and *AMBOSS* data sets: 64.4% (56/87) versus 57.8% (59/102) and 44% (44/100) versus 42% (42/100), respectively. Furthermore, the model performed better on *NBME* questions when compared to *AMBOSS* questions, for both Step 1 and Step 2: 64.4% (56/87) versus 44% (44/100) and 57.8% (59/102) versus 42% (42/100), respectively. ChatGPT outperformed both GPT-3 and InstructGPT on all data sets. InstructGPT was outperformed by 8.15% on average, whereas GPT-3 performed similarly to random chance on all question sets.

**Table 1:** The performance of the 3 large language models (LLMs) on the 4 outlined data sets.

LLM, response		*NBME*^a^*-Free-Step1* (n=87), n (%)	*NBME-Free-Step2* (n=102), n (%)	*AMBOSS-Step1* (n=100), n (%)	*AMBOSS-Step2* (n=100), n (%)
**ChatGPT^b^**					
	Correct	56 (64.4)	59 (57.8)	44 (44)	42 (42)
	Incorrect	31 (35.6)	43 (42.2)	56 (56)	58 (58)
**InstructGPT**					
	Correct	45 (51.7)	54 (52.9)	36 (36)	35 (35)
	Incorrect	42 (48.3)	48 (47.1)	64 (64)	65 (65)
**GPT-3**					
	Correct	22 (25.3)	19 (18.6)	20 (20)	17 (17)
	Incorrect	65 (74.7)	83 (81.4)	80 (80)	83 (83)

^a^NBME: National Board of Medical Examiners.

^b^ChatGPT: Chat Generative Pre-trained Transformer.

### Question Difficulty and Model Accuracy

From Table 2, relative to AMBOSS users as reported on the after-test summary, ChatGPT was in the 30th percentile on Step 1 questions without the *Attending Tip* and the 66th percentile on Step 1 questions with the *Attending Tip*. On the Step 2 *AMBOSS* data set with and without the *Attending Tip*, the model performed at the 20th and 48th percentiles, respectively. On Step 1 questions without the *Attending Tip*, ChatGPT had a significant decrease in accuracy as the AMBOSS-reported difficulty increased (*P*=.01), falling from 64% (9/14) accuracy on level 1 questions to 0% (0/9) accuracy on level 5 questions. The remaining groups were monotonically decreasing in accuracy as question difficulty increased, except for questions with difficulty 2 versus 3 for Step 1 with the *Attending Tip* and questions with difficulty 4 versus 5 for Step 2 without the *Attending Tip*.

**Table 2:** ChatGPT’s^a^ performance on AMBOSS-Step1 and AMBOSS-Step2 data sets by question.

Step, tip, response				Overall, n (%)		Question difficulty, n (%)						*P* value
					1		2	3	4	5		
**Step 1 (overall: n=100; difficulty 1: n=14; difficulty 2: n=27; difficulty 3: n=32; difficulty 4: n=18; difficulty 5: n=9)**												
	**Without *Attending Tip***											
		Correct	44 (44)		9 (64.3)		16 (59.3)	13 (40.6)	6 (33.3)	0 (0)	.01	
		Incorrect	56 (56)		5 (35.7)		11 (40.7)	19 (59.4)	12 (66.7)	9 (100)		
	**With *Attending Tip***											
		Correct	56 (56)		10 (71.4)		16 (59.3)	21 (65.6)	7 (38.9)	2 (22.2)	.06	
		Incorrect	44 (44)		4 (28.6)		11 (40.7)	11 (34.4)	11 (61.1)	7 (77.8)		
**Step 2 (overall: n=100; difficulty 1: n=25; difficulty 2: n=23; difficulty 3: n=27; difficulty 4: n=16; difficulty 5: n=9)**												
	**Without *Attending Tip***											
		Correct	42 (42)		15 (60)		10 (43.5)	11 (40.7)	3 (18.8)	3 (33.3)	.13	
		Incorrect	58 (58)		10 (40)		13 (56.5)	16 (59.3)	13 (81.2)	6 (66.7)		
	**With *Attending Tip***											
		Correct	53 (53)		17 (68)		15 (65.2)	12 (44.4)	7 (43.8)	2 (22.2)	.08	
		Incorrect	47 (47)		8 (32)		8 (34.8)	15 (55.6)	9 (56.2)	7 (77.8)		

^a^ChatGPT: Chat Generative Pre-Trained Transformer.

### Qualitative Breakdown of Responses

Finally, in Table 3, we evaluated ChatGPT’s answer quality across 3 metrics as outlined above: presence of logical reasoning, internal information, and external information. We found that every response provided by ChatGPT provided a logical explanation of its answer selection, independent of the correctness of the response. Additionally, across both *NBME-Free-Step1* and *NBME-Free-Step2* data sets, for both correct and incorrect responses, ChatGPT used information internal to the question in 96.8% (183/189) of questions. There was no significant difference between the presence of internal information between correct or incorrect responses for either Step 1 or Step 2 data sets (*P*=.25 and *P*=.07, respectively). Finally, information external to the question was used in 92.9% (52/56) of correct responses and 48.4% (15/31) of incorrect responses for the Step 1 data set (difference of 44.5%; *P*<.001). For the Step 2 data set, external information was used in 89.8% (53/59) of correct answers and 62.8% (27/43) of incorrect answers (difference of 27%; *P*=.001). For both Step 1 and Step 2, logical errors were the most common, followed by information errors. Few statistical errors were present for either data set.

**Table 3:** Qualitative analysis of ChatGPT’s^a^ response quality for NBME^b^-Free-Step1 and NBME-Free-Step2.

Metric		*NBME-Free-Step1*			*NBME-Free-Step2*		
		Overall (n=87), n (%)	Correct (n=56), n (%)	Incorrect (n=31), n (%)	Overall (n=102), n (%)	Correct (n=59), n (%)	Incorrect (n=43), n (%)
**Logical reasoning**							
	True	87 (100)	56 (100)	31 (100)	102 (100.0)	59 (100)	43 (100)
	False	0 (0)	0 (0)	0 (0)	0 (0)	0 (0)	0 (0)
**Internal information**							
	True	84 (96.6)	55 (98.2)	29 (93.5)	99 (97.1)	59 (100)	40 (93)
	False	3 (3.4)	1 (1.8)	2 (6.5)	3 (2.9)	0 (0)	3 (7)
**External information**							
	True	67 (77)	52 (92.9)	15 (48.4)	80 (78.4)	53 (89.8)	27 (62.8)
	False	20 (23)	4 (7.1)	16 (51.6)	22 (21.6)	6 (10.2)	16 (37.2)
**Reason for incorrect answer**							
	Logical error	—^c^	—	13 (41.9)	—	—	16 (37.2)
	Information error	—	—	7 (22.6)	—	—	13 (30.2)
	Statistical error	—	—	2 (6.5)	—	—	1 (2.3)
	Logical and information errors	—	—	9 (29)	—	—	13 (30.2)

^a^ChatGPT: Chat Generative Pre-Trained Transformer.

^b^NBME: National Board of Medical Examiners.

^c^Not applicable.

## Discussion

### Principal Findings

One of the key features touted by the advancement of ChatGPT is its ability to understand context and carry on a conversation that is coherent and relevant to the topic at hand. In this paper, we have shown that this extends into the medical domain by evaluating ChatGPT on 4 unique medical knowledge competency data sets, framing conversation as question answering. We found that the model is capable of correctly answering up to over 60% of questions representing topics covered in the USMLE Step 1 and Step 2 licensing exams. A threshold of 60% is often considered the benchmark passing standards for both Step 1 and Step 2, indicating that ChatGPT performs at the level expected of a third-year medical student. Additionally, our results demonstrate that even in the case of incorrect answers, the responses provided by the model always contained a logical explanation for the answer selection, and greater than 90% of the time, this response directly included information contained in the question stem. Correct answers were found to contain information external to the question stem significantly more frequently (given a threshold of *P*<.001 [19]) than incorrect responses, indicating that the ability of the model to correctly answer a question may be related to its ability to relate the prompt to data within its armamentarium.

Prior work in the field of medical question answering research has often been focused on more specific tasks with the intent of improving model performance at the expense of generalizability. For example, Jin et al [20] achieved a 68.1% accuracy with their model that answers yes-or-no questions whose answers may be found in the corpus of PubMed-available abstracts. Attempts at more generalizable models have been met with more challenges. A different Jin et al [21] achieved an accuracy of 36.7% on a data set of 12,723 questions derived from Chinese medical licensing exams. Similarly, in 2019, Ha et al [22] reported only a 29% accuracy on 454 USMLE Step 1 and Step 2 questions. Expanding beyond simple question-answering tasks, ChatGPT therefore represents a significant step forward on 3 distinct fronts. First is generalizability, as ChatGPT is capable of responding to any question that can be formatted with text alone; the scope of possible questions is limited only by what can be submitted by the user. The second front is accuracy. We have shown that ChatGPT equals or outperforms prior models on questions of similar difficulty and content. Finally, ChatGPT marks the greatest jump forward in user interpretability due to its conversational interface. Each response has some level of reasoning as we have demonstrated, and the ability to ask follow-up questions allows the user to gain a larger perspective on the concept being addressed in the question, rather than just an answer output alone.

This dialogic nature is what separates ChatGPT from previous models in its ability to act as an educational tool. InstructGPT performed at an accuracy above random chance, although still below ChatGPT on all data sets. However, even if InstructGPT performed at an accuracy equal to ChatGPT, the responses InstructGPT provided were not as conducive to student education. InstructGPT’s responses were frequently only the selected answer with no further explanation, and it is impossible to ask follow-up questions to gain more context. As InstructGPT is not formatted as a dialogic system, the model will often continue the prompt rather than provide a distinct answer. For example, a prompt ending in “G) Delirium” will be extended into “tremens B) Dislodged otoliths” before an answer is provided. GPT-3 suffers from similar fallbacks and requires more prompt engineering to generate the desired output [17]. Additionally, the model performed far below both ChatGPT and InstructGPT on all data sets.

One potential use case to highlight for the use of ChatGPT is as an adjunct or surrogate for small (peer) group education. Small group education has been shown to be a highly efficacious method of teaching [23,24]. Specific examples of facilitating small group discourse in medical education include clinical problem-solving by working through case presentations [25]. Such an approach to education is useful and independent of the knowledge of the students, as evidenced by small group education starting as early as the first week after matriculation within the Yale System of Medical Education [26]. Rees et al [27] also demonstrated that students taught by peers do not have significantly different outcomes than students taught by faculty. An aspect of small group education that is often beneficial is the ability of students to test ideas off of each other and receive feedback. With its dialogic interface, ChatGPT is able to provide many of these same benefits for students when they are studying independently. Students could use the tool to ask questions about specific medical concepts, diagnoses, or treatments and receive accurate and personalized responses to help them better structure their knowledge around each concept. For example, author CWS provides the following reflection on his use of ChatGPT while reviewing particularly challenging problems from a recent virology midterm. He found value in plugging questions into ChatGPT and engaging with follow-up dialogue, because it could unearth context relevant to the question and effectively trigger recall for specific lectures that taught the material relevant to the problem. This suggests that the context that ChatGPT provides in an initial answer could open the door for further questioning that naturally digs into the foundational knowledge required to justify the given underlying medical reasoning. Further studies are needed to evaluate the specific efficacy of ChatGPT for the simulation of small group education, as well as other use cases that may be beneficial (such as the process of reflective learning) [28]. As the technology is further explored and improved, it is also possible that novel educational methods may be developed that fully use the capabilities of a tool such as ChatGPT.

### Limitations

This study has several limitations. First, ChatGPT was first trained on a corpus that was created from data produced on or before 2021. This limits the model’s prompts to contain only information found prior to that date. Second, due to the closed nature of this model and the lack of a public API, we are unable to fine-tune this model on task-specific data and examine the breadth of its inherent stochasticity. However, this work investigates ChatGPT’s in-context performance on USMLE Step 1 and 2 exams, so these limitations did not hamper our analysis. Third, ChatGPT is receiving regular updates, believed to be the result of training on inputs as they are provided by users. The version of ChatGPT used in this study was not the most up-to-date model at the time of publication. All said, it is reasonable to hypothesize that each new iteration of the model will not cause a significant reduction in model performance on the task we have outlined and, in fact, may cause an increase in performance.

### Conclusions

In conclusion, our results suggest that ChatGPT performs at a level expected of a third-year medical student on the assessment of the primary competency of medical knowledge. Furthermore, the tool has potential as an innovation within the context of small group education in medicine (such as for problem-based learning or clinical problem-solving). ChatGPT’s responses to questions provide interpretable context to justify the written response of the model in most cases, suggesting narrative coherence. By providing sufficiently accurate dialogic responses akin to human learners, the model may facilitate the creation of an on-demand, interactive learning environment for students, which has the potential to support problem-solving and externally supported reflective practice.

To provide additional value to the reader as to the functioning of ChatGPT, a second conclusion to this paper was written by the tool using the prompt “Write a conclusion for the following manuscript:” with the entire text of the manuscript up to but excluding the handwritten conclusion included in the prompt:

In conclusion, this study aimed to assess the performance of the ChatGPT language model on examinations which evaluate medical knowledge and reasoning, as well as its ability to serve as a virtual medical tutor. The study used two pairs of datasets, AMBOSS-Step1 and AMBOSS-Step2, and NBME-Free-Step1 and NBME-Free-Step2, to examine ChatGPT’s understanding of medical knowledge related to USMLE Step 1 and Step 2 Clinical Knowledge exams. The results of the study showed that ChatGPT’s performance on the medical knowledge examination was superior to that of GPT-3 and InstructGPT, and that the additional context provided by the *Attending Tip* improved its performance. Additionally, the study found that ChatGPT’s responses were coherent in terms of logical reasoning, use of internal information, and use of external information. Overall, this study suggests that ChatGPT has the potential to be used as a virtual medical tutor, but more research is needed to further assess its performance and usability in this context.

## Appendix

Multimedia Appendix 1 Spreadsheet of all questions, annotations, and ChatGPT responses for all four datasets.

## Abbreviations

API: application programming interface
ChatGPT: Chat Generative Pre-trained Transformer
LLM: large language model
NBME: National Board of Medical Examiners
USMLE: United States Medical Licensing Examination

## Data Availability

The data sets analyzed during this study are available in Multimedia Appendix 1.
