# Correction: How Does ChatGPT Perform on the United States Medical Licensing Examination (USMLE)? The Implications of Large Language Models for Medical Education and Knowledge Assessment

**DOI:** 10.2196/57594

**Published:** 2024-02-27

**Authors:** Aidan Gilson, Conrad W Safranek, Thomas Huang, Vimig Socrates, Ling Chi, Richard Andrew Taylor, David Chartash

**Affiliations:** 1 Section for Biomedical Informatics and Data Science Yale University School of Medicine New Haven, CT United States; 2 Department of Emergency Medicine Yale University School of Medicine New Haven, CT United States; 3 Program of Computational Biology and Bioinformatics Yale University New Haven, CT United States; 4 School of Medicine University College Dublin National University of Ireland, Dublin Dublin Ireland

In “How Does ChatGPT Perform on the United States Medical Licensing Examination (USMLE)? The Implications of Large Language Models for Medical Education and Knowledge” (MIR Med Educ 2023;9:e45312) three additions were made to enhance discoverability.

The title originally appeared as:

*How Does ChatGPT Perform on the United States Medical Licensing Examination? The Implications of Large Language Models for Medical Education and Knowledge Assessment*

And has been changed to:

*How Does ChatGPT Perform on the United States Medical Licensing Examination (USMLE)? The Implications of Large Language Models for Medical Education and Knowledge*

In the “Objective” section of the Abstract, the following sentence:

*This study aimed to evaluate the performance of ChatGPT on questions within the scope of the United States Medical Licensing Examination Step 1 and Step 2 exams, as well as to analyze responses for user interpretability.*

Has been changed to read as:

*This study aimed to evaluate the performance of ChatGPT on questions within the scope of the United States Medical Licensing Examination (USMLE) Step 1 and Step 2 exams, as well as to analyze responses for user interpretability.*

Finally, the abbreviation “USMLE” has been added to the Keywords section.

The correction will appear in the online version of the paper on the JMIR Publications website on February 27, 2024 together with the publication of this correction notice. Because this was made after submission to PubMed, PubMed Central, and other full-text repositories, the corrected article has also been resubmitted to those repositories.

